# Interferon-induced transmembrane proteins: membrane gatekeepers of fusion and infection

**DOI:** 10.3389/fcimb.2026.1794791

**Published:** 2026-05-08

**Authors:** Melika Amoueian, Qian Liu, Lucienne Tritten

**Affiliations:** 1 Institute of Parasitology, McGill University, Sainte-Anne-de-Bellevue, QC, Canada; 2 Mark Wainberg Center for Viral Diseases, Lady Davis Institute, Montreal, QC, Canada; 3 Department of Biochemistry, McGill University, Montreal, QC, Canada

**Keywords:** extracellular vesicles, host-pathogen arms race, IFITM-1, IFITM-2, IFITM-3, interferon-induced transmembrane proteins (IFITMs), membrane fusion blockade, post-translational modifications

## Abstract

Interferon-induced transmembrane proteins (IFITMs) are small, non-enzymatic factors that reshape host cell membranes to block pathogen entry. In this review, we bring together what is known about their genetics and evolution, how their structure relates to function, and how they are regulated after translation. A central theme is how the amphipathic helix organizes lipids and bends membranes to stop fusion pores from forming. We connect well-studied antiviral cases, influenza A, HIV-1, flaviviruses, and the context-dependent effects seen with coronaviruses, to newer cell-biology insights, including cooperation with ZMPSTE24 and dependence on phosphoinositides. Beyond viruses, IFITMs appear to control how extracellular vesicles deliver cargo, acting as broader “membrane gatekeepers” of cell-to-cell communication. Placing IFITMs in the host–pathogen arms race, we outline how viruses evade IFITM activity or dampen its induction, while helminth and protozoan parasites rewire interferon pathways more broadly; their secreted miRNAs and proteases commonly suppress NF-κB and may intersect with IFITM-regulated uptake. Finally, we survey noncanonical roles in development, immunity, and cancer, and highlight open questions about topology, lipid dynamics, and targeting specificity. Together, these threads present IFITMs as adaptable effectors linking innate immunity, membrane biophysics, and disease, with clear implications for antivirals, immuno-oncology, and vesicle-based therapies.

## 1 Introduction

The interferon (IFN) system provides the first line of defense against infection and coordinates the induction of hundreds of interferon-stimulated genes (ISGs) that collectively produce an antiviral state (Schneider et al., 2014; Schoggins, 2019). Interferon-induced transmembrane proteins (IFITMs) have emerged as some of the most diverse and widespread restriction factors. IFITMs are small and non-enzymatic, but they can dramatically alter the properties of the host membrane and create an environment that prevents the fusion of viral and cellular membranes (Friedlová et al., 2022; Gómez-Herranz et al., 2023). IFITMs were formally identified as broad viral restriction factors in 2009 through genome-wide screens, they have since been extensively studied in viruses such as influenza A, human immunodeficiency virus type 1 (HIV-1), flaviviruses, and coronaviruses, where they play a key role in innate immunity (Brass et al., 2009; Everitt et al., 2012; Savidis et al., 2016).

The biology of IFITMs extends beyond viral entry blockade. Their activity is shaped by post-translational regulation, and their diversification across vertebrate lineages reflects gene-family expansion and lineage-specific adaptation rather than strict one-to-one functional equivalence between species (Zhang et al., 2012; Compton et al., 2016). Depending on the context, IFITMs can take on unexpected roles such as contributing to cancer progression, influencing cell differentiation, or even enhancing infection in some settings (Prelli Bozzo et al., 2021; Chu et al., 2022). More recently, they have been implicated in regulating communication via extracellular vesicles (EVs), expanding their significance well beyond virology (Bonsergent et al., 2021; Kelemen et al., 2021).

This review summarizes current knowledge of IFITM 1-3, the best-characterized antiviral members of the IFITM family, from their molecular features to their role as barriers to membrane fusion and interactions with diverse pathogens. We highlight open questions, future directions, and emerging roles in parasitic infections, underscoring their potential as therapeutic targets. Together, these perspectives position IFITMs at the crossroads of innate immunity, pathogen evolution, and human disease.

## 2 The IFITM family - molecular and cellular fundamentals

### 2.1 Discovery, genetics, and evolution

The *IFITM* genes were first identified in 1984, when researchers screening human glioblastoma T98G cells detected transcripts that were strongly induced by interferon-α (IFN-α) treatment. The transcripts, initially named 9-27 (Leu-13), 1-8D, and 1-8U, were subsequently designated as *IFITM1*, *IFITM2*, and *IFITM3*, respectively (Friedman et al., 1984; Sällman Almén et al., 2012). These genes were first characterized for their response role to IFN with the mechanism that was called interferon-stimulated response element (ISRE)-mediated regulation (Lewin et al., 1991; Hickford et al., 2012). An antiviral effect of the interferon-induced protein 9-27 (now IFITM1) was first observed in 1996, when Alber and Staeheli showed that its overexpression partially inhibited replication of vesicular stomatitis virus (VSV) (Alber and Staeheli, 1996). This antiviral function was further demonstrated in 2009, when genome-wide siRNA screens in human cells identified IFITM1–3 as host restriction factors for influenza A virus (IAV) with validation in primary human cells and *Ifitm*-deficient mouse embryonic fibroblasts (Brass et al., 2009). In humans, the IFITM loci comprise five members: *IFITM1, IFITM2, IFITM3, IFITM5* (also known as BRIL, bone-restricted IFITM-like (Maranda et al., 2022)), and *IFITM10*. Four genes (*IFITM1, IFITM2, IFITM3*, and *IFITM5*) reside within a ~26 kb segment on chromosome 11p15.5, while *IFITM10* is located about 1.4 Mb further away from this cluster (Ren et al., 2020). The murine genome encodes seven *Ifitm* genes; six form a syntenic cluster on chromosome 7, despite *Ifitm7* being located separately on chromosome 16 (Liao et al., 2019). All human and mouse gene members contain one intron and two exons, apart from *Ifitm7* (Ren et al., 2020). Phylogenetic comparisons show that IFITM homologues are present in diverse vertebrate groups, including fish, reptiles, birds, and mammals, highlighting their deep evolutionary origin and conserved role in host defense (Zhang et al., 2012; Hickford et al., 2012; Schelle et al., 2023). Tandem duplications, along with the absence of strict one-to-one orthologs between mouse and human *IFITMs*, suggest that these genes have undergone lineage-specific expansions and follow separate evolutionary paths (Bailey et al., 2012). Notably, members of the immune-associated subfamily *IFITM1–3* show signatures of positive selection, consistent with adaptive evolution driven by repeated pathogen pressures (Friedlová et al., 2022).

In vertebrates, *IFITM* genes originate from three progenitors forming three distinct clades: Clade I: IR-*IFITM* (Immunity-Related genes like *IFITM1, 2, 3, 6, 7*), Clade II: *IFITM5* (Bone-related), Clade III: *IFITM10* (Function unknown) (Hickford et al., 2012). *IFITM2* (1-8D) and *IFITM3* (1-8U) are extremely similar (>90%) in both their protein-coding and regulatory regions. This suggests a very recent gene duplication event. In contrast, *IFITM1* (9-27) is different in its regulatory regions (Lewin et al., 1991; Friedlová et al., 2022). *IFITM3* is strongly induced by IFN due to its functional promoter elements, while *IFITM2* is only weakly induced because these same elements contain mutations (Lewin et al., 1991). Lacking a standard TATA box, *IFITM* gene promoters likely use an alternative “initiator” element, revealing that the IFN signaling pathway is versatile enough to activate genes with different promoter structures (Lewin et al., 1991). In primates, *IFITM5* and *IFITM10* are conserved single-copy orthologs with high sequence conservation, whereas the immunity-related *IFITMs* show marked copy-number and sequence diversification across lineages (Schelle et al., 2023). The IR-*IFITM* clade shows rapid and dynamic evolution driven by a “birth-and-death” model, where they are frequently duplicated and lost in response to pathogens, providing strong evolutionary evidence consistent with their antiviral roles (Zhang et al., 2012). While the bone-specific function of *IFITM5* is a recent adaptation in therian mammals, the gene itself is highly conserved and shows no signs of positive selection. This specificity, however, presents a scientific puzzle, as knockout mouse models reveal its absence causes only minor and transient skeletal defects, suggesting a non-essential role for normal bone development. In other vertebrates like fish and birds, *IFITM5* is expressed in tissues like the brain and liver, but not bone (Hickford et al., 2012; Patoine et al., 2017). The biological function of *IFITM10* remains unknown; however, evidence of gene duplication and positive selection in aquatic vertebrates suggests it may have contributed to adaptation to aquatic environments (Zhang et al., 2012). *IFITM5* and *IFITM10* have clear and direct evolutionary orthologs between species. *IFITM10* gene has a highly conserved sequence but its expression pattern is completely different in mice (brain and spleen) compared to humans (bone) and marsupials (bone), creating a paradox here (Hickford et al., 2012).

### 2.2 Structure, topology, and localization

IFITM proteins are approximately 17 kDa (corresponding to ~130 amino acids) and are commonly grouped within the Dispansin/CD225 superfamily on the basis of sequence and domain organization, although their precise membrane topology remains debated (Ren et al., 2020; Xie et al., 2024). Functional *IFITM* genes encode proteins with a conserved CD225 domain, located within the membrane. The proteins are generally small and consist of a long N-terminal domain (NTD), two hydrophobic intramembrane/transmembrane domains (IM1 and IM2), a conserved cytoplasmic/intracellular loop (CIL), and a short C-terminal domain (CTD) (Jia et al., 2012; John et al., 2013). The CD225 domain itself is composed of the IM1 domain and the CIL (John et al., 2013).

**Figure 1:**
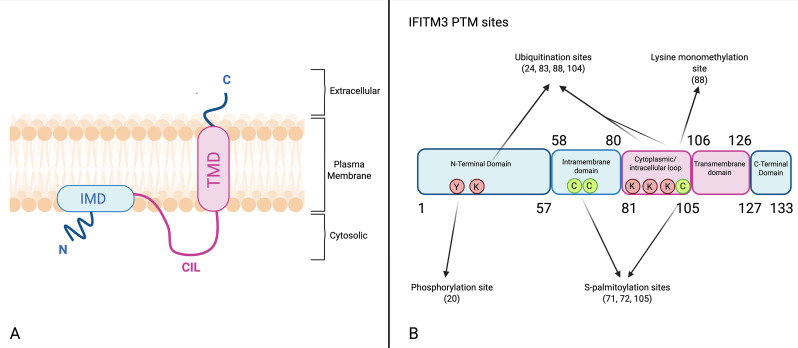
**(A)**. Proposed membrane topology of IFITM3. Schematic representation of IFITM3 showing its two hydrophobic regions: the intramembrane domain (IMD) and the transmembrane domain (TMD). The N-terminus (N) is cytoplasmic, while the C-terminus (C) faces the extracellular or luminal side, consistent with a type II transmembrane topology. The cytoplasmic/intracellular loop (CIL) connects the two hydrophobic regions and contains regulatory residues important for post-translational modification and antiviral function. **(B)** IFITM3 domain organization and post-translational modification sites. Linear schematic of IFITM3 showing the N-terminal domain (NTD; residues 1-57), intramembrane domain (IMD; residues 58-80), cytoplasmic/intracellular loop (CIL; residues 81-105), transmembrane domain (TMD; residues 106-126), and C-terminal domain (CTD; residues 127-133), together with annotated post-translational modification sites. Phosphorylation occurs at Y20; S-palmitoylation occurs at C71 and C72 within the IMD and at C105 at the membrane-proximal end of the CIL; ubiquitination occurs at K24, K83, K88, and K104; and lysine monomethylation occurs at K88. These modifications collectively regulate IFITM3 localization, stability, and antiviral activity. Created with BioRender.com.

IFITM1, IFITM2 and IFITM3 share a conserved domain architecture and membrane-associated topology, although IFITM3 has been the most extensively characterized (Bailey et al., 2014). The most widely supported model for IFITM3 is a Type II transmembrane topology, with an intracellular (cytoplasmic) N-terminus and an extracellular (luminal) C-terminus (**Figure 1**). The N-terminal region exhibits a predominantly flexible, random coil structure (Bailey et al., 2013; Xie et al., 2024). This model consists of an intramembrane domain (M1) that is partially buried in the inner leaflet of the membrane without fully crossing it, and a second, long α-helix (M2) that forms a single continuous transmembrane domain, placing the C-terminus outside the cell. M1’s intramembrane positioning may promote membrane curvature (Ling et al., 2016).

IFITM1 shares high homology with IFITM3 and predominantly adopts a type II membrane topology, with a cytoplasmic N-terminus and an extracellular C-terminus (Bailey et al., 2014).

Because IFITM3 is crucial for restricting IAV *in vivo*, it is the most intensively studied family member and is commonly used as a topology model (Everitt et al., 2012). Its N-terminal domain (amino acids 1-57) is characterized by sequence diversity and hydrophobicity, features that are important for its localization and function (Ren et al., 2020). This domain is a critical regulatory platform that contains key motifs that determine protein trafficking and stability. The extended N-termini of IFITM2 and IFITM3 contain a tyrosine-based sorting motif (YxxΦ, where Φ is a bulky hydrophobic amino acid) and a PPxY motif that serves as a docking site for the E3-ubiquitin ligase NEDD4 (Chesarino et al., 2014a). These motifs are pivotal in regulating endocytosis and protein turnover. A key structural distinction within the family is that IFITM1 lacks this extended N-terminus, a difference that directly contributes to its distinct localization at the plasma membrane compared to the endosomal localization of IFITM2 and IFITM3 (Jia et al., 2012; Chesarino et al., 2014a; Marziali et al., 2021).

Downstream of the NTD is a conserved hydrophobic domain that contains 22 amino acids (residues 58–80 in IFITM3) (Chesarino et al., 2014a). This region is understood to be an intramembrane domain instead of a full transmembrane helix. It does not fully span the lipid bilayer but instead inserts into the cytosolic leaflet. It contains a highly conserved, short amphipathic helix (AH, 59-68), which is the key driver of IFITM’s antiviral activity (Chesarino et al., 2014a; Zhao et al., 2018). The amphipathic helix directly stiffens and reshapes cellular membranes by increasing lipid order, reflected by higher Laurdan GP (generalized polarization) and reduced fluidity, and by promoting or stabilizing positive curvature at fusion intermediates. Together, these effects block fusion pore formation and viral entry (Li et al., 2013; Chesarino et al., 2017; Guo et al., 2021; Rahman et al., 2020). Within this domain, two specific phenylalanine residues, F75 and F78, are essential for IFITM3’s antiviral activity. Early studies proposed that these residues mediate IFITM3 oligomerization (John et al., 2013). However, later work showed that an IFITM3 mutant carrying substitutions at F75 and F78 lost antiviral activity while retaining IFITM3-IFITM3 interactions (Winkler et al., 2019). This indicates that while F75 and F78 are indispensable for viral restriction, their role does not lie in promoting oligomerization, but likely in affecting local structure or membrane interactions critical for IFITM3 function.

A conserved intracellular loop, spanning residues 81 to 105 in IFITM3, connects the intramembrane domain to the second hydrophobic region. This loop contains several lysine residues that serve as ubiquitination sites and includes the membrane-proximal cysteine C105, which undergoes S-palmitoylation. Together, these features highlight the importance of this region in regulating IFITM3 stability and function (Yount et al., 2010; Jia et al., 2012; Yount et al., 2012; Tiwari and Viswanath, 2022). The second hydrophobic region, spanning residues 106 to 126, is structurally distinct from the IMD and forms a true transmembrane helix that crosses the lipid bilayer (Bailey et al., 2013; Ling et al., 2016). The protein terminates with a short and highly variable C-terminal domain (residues 127–133 in IFITM3) (Bailey et al., 2014; Ling et al., 2016). This structural diversity among IFITM family members underlies specific regulatory roles and specific transport signals. For example, the CTD in IFITM1 contains a basic KRXX motif that regulates the protein’s distribution between endosomes and the plasma membrane by binding to the AP-3 adaptor complex. Mutation or deletion of this region alters the protein’s location and modulates its antiviral activity in a virus-dependent manner (Li et al., 2015).

The antiviral activity of IFITM proteins is dynamic and tightly regulated by multiple post-translational modifications (PTMs) (**Figure 1**) (Chesarino et al., 2017; Wen et al., 2022). These modifications act like molecular switches, finely tuning the protein’s localization, stability, and function (Yount et al., 2012). The most studied member of the family, IFITM3, is regulated by at least four known types of PTMs: S-palmitoylation, phosphorylation (e.g., Y20), ubiquitination (e.g., K24, K83, K88, K104), and lysine methylation. Together, these PTMs act as a rheostat rather than a simple on/off switch (Wen et al., 2022). S-palmitoylation is the only known PTM that enhances IFITM3 antiviral activity by anchoring it to membranes and improving its function. In contrast to S-palmitoylation, phosphorylation, ubiquitination, and methylation act as regulatory “brakes”, controlling intracellular trafficking, stability, and membrane interactions (Chesarino et al., 2014b; Tiwari and Viswanath, 2022; Wen et al., 2022). These modifications are not absolute inhibitors but act in a context-dependent manner. They can weaken IFITM3 activity, change its location within the cell, or alter which viruses it can restrict. As a result, the final antiviral state of a cell is the result of a balance between these opposing yet cooperative changes (Chesarino et al., 2014b; Wen et al., 2022; Unali et al., 2023).

S-palmitoylation is the reversible attachment of a 16-carbon fatty acid, palmitate, to cysteine residues. It is the only positive post-translational modification for IFITM3 function (Bailey et al., 2014; McMichael et al., 2017; Yuan et al., 2024). This modification is processed by members of the ZDHHC family of palmitoyltransferases. While multiple ZDHHCs can palmitoylate IFITM3, ZDHHC20 plays a prominent role because it colocalizes with IFITM3 in lysosomes and enhances its antiviral activity (McMichael et al., 2017). Three conserved cysteines (C71, C72 within the intramembrane domain, and C105 in the cytoplasmic loop) are the main S-palmitoylation sites and play a crucial role in maintaining antiviral activity (Yount et al., 2010). S-palmitoylation is a potent antiviral PTM of IFITM3, critical for restricting a wide range of viruses, including IAV and Ebola virus; disrupting this modification significantly reduces the protein’s antiviral activity (Das and Hang, 2023).

The phosphorylation of IFITM3 acts as a key trafficking switch, dynamically changing its subcellular localization and its antiviral specificity (Compton et al., 2016). This modification occurs at tyrosine 20 (Y20) within the N-terminal domain. Y20 is a key part of a standard YxxΦ endocytic sorting motif. This motif is recognized by the adaptor protein complex AP-2, which mediates internalization of IFITM3 from the plasma membrane into endosomes (Chesarino et al., 2014a; Friedlová et al., 2022). Phosphorylation of IFITM3 at Y20 by the kinase Fyn interferes with AP-2-mediated endocytic sorting, leading to its accumulation at the plasma membrane. Mutation or phosphorylation of Y20 reduces ubiquitination of IFITM3, increasing its stability, but also alters its localization (Chesarino et al., 2014a; Friedlová et al., 2022). In experiments from Chesarino et al. (2014), mutation of Y20 impairs IFITM3’s antiviral activity against IAV. The impact of Y20 alteration on HIV-1 restriction is less well defined in published studies to date (Chesarino et al., 2014b).

Ubiquitination generally acts as a negative regulator of IFITM3. E3 ubiquitin ligases (notably NEDD4) catalyze the addition of ubiquitin to multiple lysine residues of IFITM3, promoting its endocytic internalization and lysosomal degradation (Chesarino et al., 2014a; Chesarino et al., 2015). Reduced levels of IFITM3 impair antiviral activity (i.e., increase viral susceptibility). Interestingly, phosphorylation at Y20 by Fyn inhibits ubiquitination (by blocking the YXXΦ endocytic sorting signal), thus indirectly stabilizing IFITM3 by reducing its internalization and degradation (Chesarino et al., 2014a).

Lysine monomethylation of IFITM3 at K88, installed by SET7, provides another regulatory layer that dampens antiviral activity (Shan et al., 2013). Importantly, this modification is reversible: during IFN signaling, the demethylase LSD1 removes the methyl group and restores IFITM3’s antiviral function (Shan et al., 2013). Viral infections such as IAV and VSV promote SET7–IFITM3 interactions, driving K88 methylation and weakening host defenses, whereas IFN signaling counteracts this by recruiting LSD1. Although the precise mechanism by which K88 methylation downregulates IFITM3 remains unresolved, it exemplifies viral subversion of host PTM pathways to dampen innate immunity (**Table 1**) (Shan et al., 2013; Zani and Yount, 2018).

**Table 1:** Post-translational modifications (PTMs) of human IFITM3.

Modification	Residue(s)	Key enzyme(s)	Regulatory effect / mechanism
S-Palmitoylation	Cys71, Cys72, Cys105	zDHHC(s), especially ZDHHC20	Palmitoylation enhances IFITM3 antiviral activity by anchoring its amphipathic helices, promoting membrane clustering (formation of IFITM3-enriched microdomains), and stabilizing interactions with sterols. Loss of palmitoylation greatly reduces activity.
Phosphorylation	Tyr20	Fyn Kinase	Negative regulation (at least in certain contexts / for some viruses), phosphorylation reduces activity against endosomal viruses by altering localization: it blocks binding of AP-2, prevents endocytosis (or alters internalization) leading to altered localization (plasma membrane vs endosomes).
Ubiquitination	Lys24, Lys83, Lys88, Lys104	E3-ubiquitin ligase NEDD4	Negative regulation: ubiquitination marks IFITM3 for degradation (proteasomal/lysosomal), thus controlling steady-state levels; mutations of these sites (e.g. to alanine) reduce ubiquitination and increase stability / antiviral activity.
Methylation	Lys88	SET7 (methyltransferase) / LSD1 (demethylase)	Negative regulation: methylation at K88 reduces antiviral function; demethylation by LSD1 restores/enhances function. Mechanism not fully resolved.

This table summarizes the four major PTMs that regulate IFITM3, detailing the specific amino acid residues targeted, the key enzymes involved in their addition or removal, as well as the overall regulatory impact on antiviral function, and the underlying molecular mechanisms (Bailey et al., 2014; Chesarino et al., 2015; Chesarino et al., 2014b; Friedlová et al., 2022; McMichael et al., 2017; Shan et al., 2017, 2013; Yount et al., 2010, 2012).

## 3 Gatekeepers of membrane fusion

### 3.1 The biophysical blockade of membrane fusion

**Figure 2:**
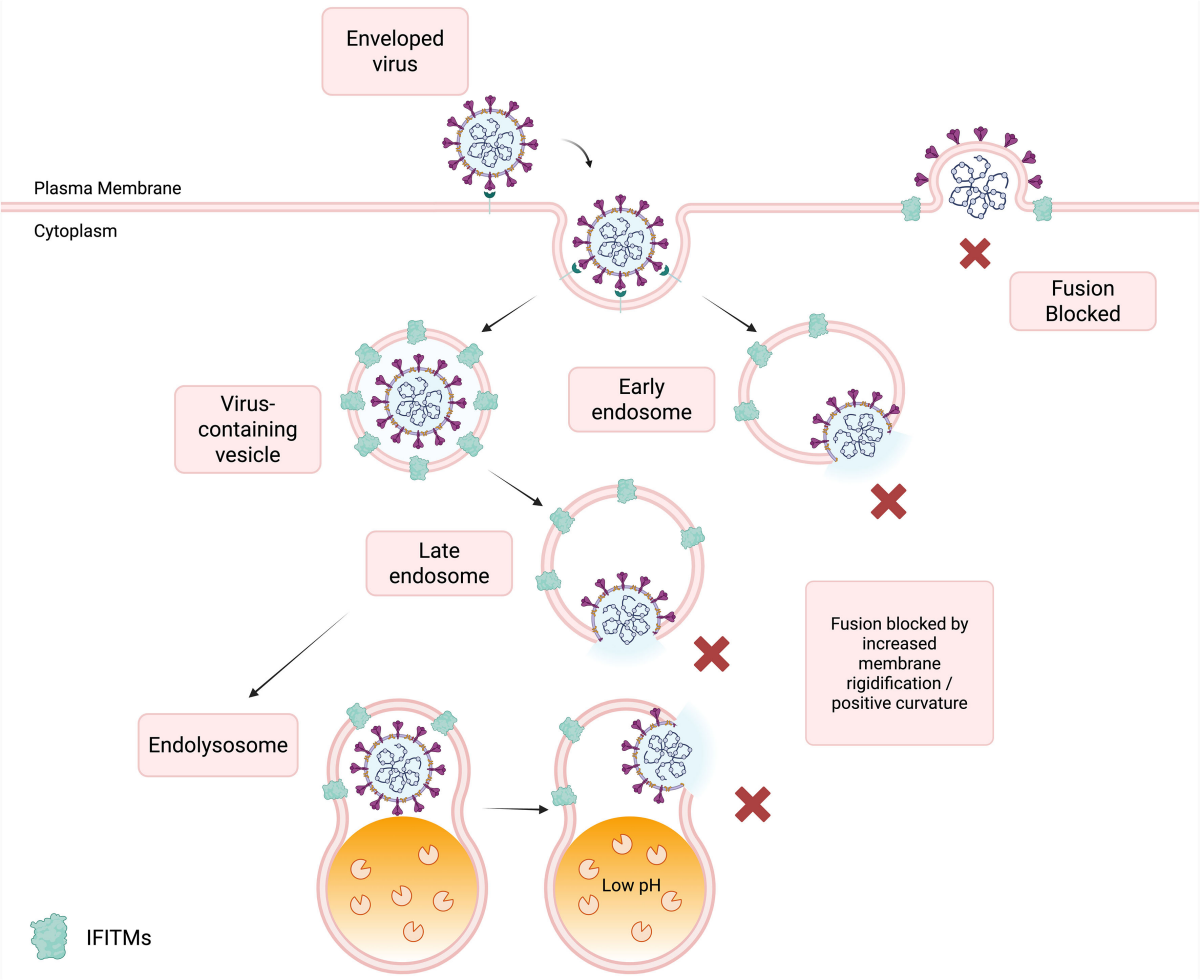
Fusion blockade during endocytic entry. Schematic of enveloped-virus entry by plasma membrane fusion or endocytic uptake into early and late endosomes, followed by trafficking to acidic endolysosomal compartments. IFITM proteins (green) are shown on plasma and endosomal membranes, where they inhibit productive membrane fusion and block fusion-pore formation without preventing endocytosis. Low pH indicates the acidic lumen of endolysosomal compartments. Increased membrane rigidification and positive curvature are proposed to contribute to this fusion blockade. (Created in BioRender; concept based on Brass et al., 2009; Amini-Bavil-Olyaee et al., 2013; Desai et al., 2014; Suddala et al., 2019.).

IFITMs function as membrane gatekeepers, establishing a biophysical barrier to viral entry by shifting membrane fluidity and curvature into an antiviral state. In IFITM-expressing cells, Laurdan fluorescence imaging shows elevated generalized polarization values and prolonged lifetimes, indicating increased lipid packing (i.e. reduced membrane fluidity) (Li et al., 2013). IFITM3 is proposed to induce positive spontaneous curvature in the cytosolic leaflet of the bilayer, a geometry thermodynamically unfavorable for hemifusion and viral membrane merger (Li et al., 2013; Rahman et al., 2020). Together, these changes create a blockade at the virus–cell interface, stalling fusion at intermediate steps (Bailey et al., 2013; Chesarino et al., 2017; Wang et al., 2024). These effects stall fusion at late intermediates in compartments consistent with the known localization of individual IFITM family members (**Figure 2**).

By reinforcing a biophysical barrier, IFITMs also serve as scaffolds that spatially organize other proteins and lipids to produce a rigidified membrane environment (Majdoul and Compton, 2022). For instance, IFITM1, IFITM2, and IFITM3 bind vesicle-associated membrane protein-associated protein A (VAPA) and prevent its canonical interaction with the oxysterol-binding protein (OSBP), thereby disrupting VAPA–OSBP–mediated cholesterol transfer at endoplasmic reticulum-endosome contact sites (Amini-Bavil-Olyaee et al., 2013). This perturbation leads to cholesterol accumulation in multivesicular bodies and late endosomes, which in turn interferes with membrane fusion processes (Amini-Bavil-Olyaee et al., 2013). Using direct virus-cell fusion assays and single-virus imaging in live cells, Desai et al. (2014) revealed that IFITM3 does not inhibit lipid mixing (hemifusion) but rather blocks the transition from hemifusion to full fusion by arresting fusion-pore formation (i.e. a post-hemifusion block) (Desai et al., 2014). In other words, the remodeling of cholesterol distribution sets up a less permissive membrane state, but the critical antiviral effect of IFITM3 lies in its ability to stabilize the cytoplasmic leaflet of the endosomal membrane and inhibit the opening or expansion of fusion pores. Thus, IFITM3 “armors” the endosomal membrane via both altered cholesterol homeostasis and direct stabilizing effects at the fusion interface (Amini-Bavil-Olyaee et al., 2013; Desai et al., 2014).

Another key component of the IFITM-scaffolded defense network is the zinc metalloprotease ZMPSTE24. Co-immunoprecipitation experiments have shown that ZMPSTE24 physically associates with IFITM1, IFITM2, and IFITM3. Interestingly, ZMPSTE24 is a potent and constitutively expressed restriction factor with a broad spectrum of antiviral activity. It largely resembles that of the IFITM proteins, inhibiting enveloped viruses such as IAV, Ebola, and Zika (Li et al., 2017; Shilagardi et al., 2022).

Cell-based genetic epistasis experiments identified ZMPSTE24 as a key downstream effector of the IFITM antiviral pathway: IFITM-mediated restriction requires ZMPSTE24, and its loss abolishes IFITM-dependent suppression of infection (Fu et al., 2017). ZMPSTE24 shows some antiviral activity independently of IFITMs, reinforcing its designation as a downstream effector (Shilagardi et al., 2022). Critically, ZMPSTE24 restricts viruses independently of its protease function. Active-site-deficient mutants are just as effective as the wild type in restricting virus entry. This strongly suggests that ZMPSTE24 plays a structural or organizational role within this context, rather than an enzymatic one. It is likely that the IFITM-ZMPSTE24 complex forms a higher-order protein scaffold or meshwork on the endosomal membrane. This structural barrier, combined with the altered lipid properties induced by IFITMs, forms a robust fortification that mechanically hinders close apposition of viral and cellular membranes and the conformational changes required for fusion-protein function (Fu et al., 2017; Li et al., 2017; Shilagardi et al., 2022).

### 3.2 The classic paradigm: restricting viral entry

IFITMs have been described as broad-spectrum viral restriction proteins since they were first identified as IFN-stimulated genes. Their antiviral function is most evident in viruses requiring late endosomal or lysosomal fusion. **Figure 2** summarizes the endocytic entry pathway and the stages at which host fusion restriction typically occurs. Representative pathogen groups are discussed below.

#### 3.2.1 Orthomyxoviruses: influenza A virus

IAV was first used to define IFITM restriction, and its study has been instrumental in uncovering the mechanisms by which IFITMs block viral entry (Brass et al., 2009; Bailey et al., 2012). Among the immune-associated IFITMs, IFITM3 has been referred to as the most effective and relevant inhibitor of IAV infection (Desai et al., 2014; Wang et al., 2018).

The critical role of IFITM3 in controlling IAV infection *in vivo* is supported by both animal models and human genetic studies. *In vivo*, *Ifitm3*-deficient mice show characteristic susceptibility to IAV: viral strains that are normally mild produce markedly higher lung viral titers, heightened immunopathology, and dramatically increased mortality (Everitt et al., 2012). This phenotype is also observed in human populations, where a common IFITM3 SNP, rs12252-C, creates an alternative splice site that yields a truncated protein with diminished activity (Everitt et al., 2012; Zhang et al., 2013). This allele is strongly associated with an increased risk of severe disease, hospitalization, and fatal outcomes. It provides overwhelming evidence in support of a crucial role played by IFITM3 as a major determinant of influenza severity in humans (Zhang et al., 2013; Kenney et al., 2023).

The mechanism of IAV restriction matches perfectly with the biophysical blockade model (Desai et al., 2014). IAV enters host cells through endocytosis. It relies on the low pH conditions of late endosomes to trigger structural changes in its hemagglutinin (HA) glycoprotein, driving membrane fusion (Aganovic, 2023). IFITM3 localizes primarily to late endosomes and lysosomes, positioning it to intercept viruses during entry (Feeley et al., 2011; Amini-Bavil-Olyaee et al., 2013; Suddala et al., 2019). Consistent with single-virus and structural studies, IFITM3 stalls IAV fusion *after hemifusion*: lipid mixing occurs, but the fusion pore does not open, so viral RNPs are not released into the cytosol. Early cell–cell fusion assays suggested a block at or before hemifusion, likely reflecting assay differences. The trapped virion is then routed to lysosomes for degradation, neutralizing the infection (Amini-Bavil-Olyaee et al., 2013; Li et al., 2013; Desai et al., 2014; Suddala et al., 2019; Klein et al., 2023).

#### 3.2.2 Retroviruses: HIV-1

Compared with the relatively direct blockade that IFITMs impose on IAV entry, IFITM‐mediated restriction of HIV-1 is more complex: it involves multiple IFITM family members, acts at several stages of the viral lifecycle, and is shaped by an evolutionary interplay between virus and host (Chutiwitoonchai et al., 2013; Tartour et al., 2014; Lee et al., 2018).

HIV-1’s sensitivity to IFITM restriction depends in part on how its co-receptor tropism aligns with the subcellular localization of IFITM isoforms (Foster et al., 2016; Gómez-Herranz et al., 2023; Marceau and Braibant, 2024). Strains that use CXCR4 (X4-tropic) tend to be more sensitive to endosomally localized IFITM2 and IFITM3, while CCR5 (R5-tropic) viruses often show greater susceptibility to IFITM1 at or near the plasma membrane. These patterns suggest that the virus’s entry route helps determine which IFITM isoform it is most likely to encounter. In this way, IFITMs act as evolutionary gatekeepers shaping viral tropism (Foster et al., 2016; Shi et al., 2017; Wu et al., 2017; Liao et al., 2019).

Furthermore, IFITMs not only block HIV-1 entry but also act post-entry. For instance, IFITMs significantly reduce the synthesis of viral proteins, such as Gag, Vif, and Nef, from integrated proviral DNA, thereby limiting the production of new virions (Lee et al., 2018). In addition, IFITMs continue functioning in the virus-producing cell, by physically interacting with the HIV-1 envelope glycoprotein (Env) (Yu et al., 2015), which disrupts the proteolytic processing of Env and its incorporation into budding virions (Yu et al., 2015; Verma et al., 2023), ultimately resulting in the release of progeny particles with reduced infectivity (Chutiwitoonchai et al., 2013; Tartour et al., 2014; Foster et al., 2016; Marceau and Braibant, 2024).

This multifaceted restriction imposes strong selective pressure on HIV-1, which in turn has evolved strategies to evade IFITMs. Transmitted/founder (T/F) viruses - the ones initiating infection - are often much more resistant to IFITM restriction than viruses isolated during the chronic phase of infection (Fenton-May et al., 2013; Foster et al., 2016). This observation indicates that evasion of the IFITM barrier poses a challenge on the route to productive transmission. Viral escape can arise from adaptive mutations in Env and Vpu that, among other effects, boost direct cell-to-cell transmission, a mode less susceptible to IFITM blockade (Ding et al., 2014; Yu et al., 2015).

#### 3.2.3 Flaviviridae: flaviviruses and hepatitis C virus

The Flavivirus family includes major human pathogens such as dengue virus (DENV), Zika virus (ZIKV), and West Nile virus, which are strongly restricted by the IFITMs (Diamond and Farzan, 2013; Savidis et al., 2016). Flaviviruses generally enter cells through receptor-mediated endocytosis, followed by pH-dependent fusion of the viral and endosomal membranes (Brass et al., 2009; Chmielewska et al., 2022). The specific subcellular localization of IFITMs is crucial for their effectiveness in inhibiting this process. Because flavivirus fusion typically occurs in endosomal compartments, the predominantly endosomal localization of IFITM2 and especially IFITM3 makes them particularly effective inhibitors (Chmielewska et al., 2022; Smit et al., 2011). The conserved KRRK cluster (aa 62–67) in the intracellular loop is required for IFITM1-mediated restriction of Zika and dengue viruses, consistent with a mechanism that blocks endosomal escape and reduces organelle acidification (Sun et al., 2020). For hepatitis C virus (HCV; a member of the Flaviviridae family), IFITM1 inhibits entry by localizing to hepatic tight junctions and disrupting assembly of the CD81 and occludin co-receptor complex (Wilkins et al., 2013). Some studies suggest that IFITM1 may also act at post-entry stages of HCV replication, but its predominant effect is believed to occur during entry (Narayana et al., 2015).

Several experimental studies support IFITMs’ antiviral activity against flaviviruses. Overexpression of IFITM proteins in cell lines produces robust inhibition, as was shown for tick-borne encephalitis virus (TBEV) (Chmielewska et al., 2022). In contrast, loss-of-function experiments underscore their physiological relevance: siRNA-mediated depletion of IFITM2 and IFITM3 markedly increases cellular susceptibility to DENV infection and weakens the antiviral effect of IFN treatment (Jiang et al., 2010).

More advanced gene-editing techniques like CRISPR-Cas9 have been used to create knockout cell lines to study IFITMs in a non-IFN-stimulated context (Chmielewska et al., 2022). In these studies, cells that lacked IFITM1 or IFITM3 were much more susceptible to TBEV infection. A double knockout of both IFITM1 and IFITM3 showed the highest vulnerability to the virus. The additive or synergistic effect of these proteins in host protection is highlighted by these findings (Chmielewska et al., 2022).

Between these different experimental models and various flaviviruses, including TBEV, ZIKV, WNV, and DENV, IFITM3 has consistently been identified as the strongest restriction factor (Savidis et al., 2016; Chmielewska et al., 2022). However, evidence from ZIKV infection indicates that IFITM3 is not always the sole determinant of IFN-mediated antiviral activity, as robust type I IFN-dependent restriction in A549 cells was observed even though this effect was not explained by IFITM3 alone (Gobillot et al., 2020). Its high baseline expression and effective blocking of viral replication suggest that IFITM3 may act as an important first line of defense against infection, even before the complete IFN response is mounted (Savidis et al., 2016). The importance of IFITM3 is further underscored by TBEV mutagenesis studies showing that its antiviral activity depends on specific features, including its N-terminal domain, which contains the YxxΦ endocytic sorting motif centered on Y20 and ubiquitination sites that regulate protein trafficking and stability, as well as phosphorylation sites (Y20), S-palmitoylation sites (C72), and residues needed for oligomerization (F75/F78) (Chmielewska et al., 2022).

Direct cell-to-cell transmission can bypass IFITM-mediated entry restriction by moving viral material through specialized cell-cell contacts (e.g., virological synapses), tunneling nanotubes (TNTs; actin-based membranous conduits that connect cells), or EVs (exosomes/microvesicles that shuttle viral cargo), thereby reducing exposure to neutralizing antibodies and other extracellular immune effectors (Tran et al., 2025). Studies on TBEV have shown that while IFITMs strongly inhibit infection by cell-free virus particles, their protective effect is surprisingly weaker during high-density infections where cell-to-cell spread is significant (Chmielewska et al., 2022).

#### 3.2.4 Coronaviridae: SARS-CoV-2 and other coronaviruses

The notion of IFITMs as purely antiviral is challenged by their paradoxical interaction with SARS-CoV-2. It is the virus–host interaction, rather than the clinical disease, that contradicts the conventional view. Depending on the specific entry route into the cell, IFITM1, IFITM2, and IFITM3 can restrict SARS-CoV-2 under overexpression conditions, whereas endogenous IFITM1–3 expression can support efficient infection in human lung cells *in vivo* (Prelli Bozzo et al., 2021; Shi et al., 2021).

This contrast highlights that human IFITM1–3 can exert either antiviral or effects depending on expression level and entry route (Prelli Bozzo et al., 2021; Shi et al., 2021). Such dual effects have been observed not only in coronaviruses but also in influenza, orthoflaviviruses (members of the genus *Flavivirus* within the family Flaviviridae, including dengue and Zika viruses), and filoviruses (members of the family Filoviridae, including Ebola and Marburg viruses (Brass et al., 2009; Huang et al., 2011; Desai et al., 2014; Prelli Bozzo et al., 2021; Shi et al., 2021; Xie et al., 2023). Together, these studies show that IFITM function is more nuanced and context-dependent than once thought.

Experimentally, this dual functionality seems to depend on the species of coronavirus, the IFITM protein involved, as well as the experimental conditions (Kenney et al., 2023; Xie et al., 2023). For instance, endogenous expression of IFITM1, IFITM2, and IFITM3 has been shown to boost the replication of SARS-CoV-1, SARS-CoV-2, and hCoV-OC43, while having minimal effect on hCoV-NL63, hCoV-229E, and MERS-CoV (Xie et al., 2023). In contrast, overexpression of IFITM1–3 inhibits infection by Angiotensin-converting enzyme 2 (ACE2)-tropic coronaviruses such as SARS-CoV-1, SARS-CoV-2, and hCoV-NL63, by preventing cell surface expression of the ACE2 receptor (Xie et al., 2023). This suggests that the concentration of IFITM proteins at the site of viral entry is an important determinant of their effect.

Studies on IFITMs in SARS-CoV-2 infection have produced highly variable and sometimes conflicting results (Kenney et al., 2023). These differences might be attributable to differences in cell lines, virus strains and experimental readouts (Shi et al., 2021; Xie et al., 2023). However, *in vivo* mouse models provide stronger evidence for IFITM3’s protective role against severe SARS-CoV-2 disease. *Ifitm3*^−/−^ mice show more pronounced body-weight loss and higher viral loads in the lung. This defect is also associated with increased post-infection mortality to mouse-adapted SARS-CoV-2 (Kenney et al., 2023). These findings highlight the importance of organism-level studies of IFITMs to reveal their overall effects on disease.

Experimental studies indicate that the antiviral activity of IFITMs is mediated by their ability to alter the physical properties of cell membranes, thereby reducing susceptibility to viral fusion. Unali et al. (2023) demonstrated that amphotericin B, which increases membrane fluidity, counteracts IFITM’s inhibitory effect on IAV when viruses enter via the plasma membrane (first shown by (Lin et al., 2013)), supporting the idea that membrane physical properties are central to IFITM antiviral activity. Importantly, lysine residues in the CIL of IFITM3 are essential for endosomal antiviral function but are dispensable for restriction of viruses that fuse at the plasma membrane (Unali et al., 2023).

ZMPSTE24 overexpression restricts SARS-CoV-2 pseudovirus infection to a degree comparable to IFITMs and, like IFITMs, modulates Spike–ACE2-mediated cell-to-cell fusion (Shilagardi et al., 2022). Notably, the catalytic activity of ZMPSTE24 is not required for its antiviral function; instead, this factor may act as a structural protein or scaffold in the complex (Shilagardi et al., 2022).

The endosomal antiviral activity of IFITM3 depends on the presence of specific phospholipids, particularly PIP3, which binds IFITM3 and is required for restriction of viruses that enter via endosomes, including SARS-CoV-2 (Unali et al., 2023).

Coronaviruses may counteract or even co-opt IFITM-mediated restriction. The evolution of SARS-CoV-2 variants of concern (VOCs) provides an example of this host-virus arms race. Early-lineage SARS-CoV-2 strains are sensitive to inhibition by guanylate-binding proteins (GBPs) 2 and 5, IFN-inducible restriction factors that interfere with Spike S1/S2 cleavage, biasing entry toward endosomes where IFITM2/3 act. Notably, Mesner et al. (2023) found that despite GBP2/5 inhibition of Spike cleavage, early-lineage isolates remained sensitive to plasma-membrane IFITM1 but not endosomal IFITM2 or IFITM3, consistent with a preference for TMPRSS2-dependent, plasma membrane entry. The Alpha and Delta variants show resistance to this GBP-mediated restriction. This resistance likely arises from adaptive mutations that enhance viral infectivity and promote immune evasion (Mesner et al., 2023). In particular, the D614G substitution in the Spike protein enhances infectivity and allows the virus to overcome the inhibitory effects of GBPs (Mesner et al., 2023).

The Omicron variant, a later lineage, is sensitive again to GBP-mediated inhibition despite carrying the D614G substitution, suggesting that additional Spike mutations compromise the ability of D614G to confer resistance (Mesner et al., 2023). Omicron shows renewed sensitivity to GBP2/5 and is also sensitive to IFITM1, IFITM2, and IFITM3; spike changes that shift entry toward TMPRSS2-independent/endosomal pathways contribute to sensitivity to endosomal IFITM2/3 (Mesner et al., 2023). Together, these findings highlight how Spike adaptations can shape viral sensitivity to multiple IFN-induced restriction factors, including both GBPs and IFITMs.

Conversely, certain mutations in IFITM1 and IFITM3 can switch them from inhibitors to enhancers of SARS-CoV and MERS-CoV entry (Zhao et al., 2018). This shows that IFITMs under specific conditions rather promote membrane fusion and facilitate viral entry (Zhao et al., 2018). The CTD of IFITM1 appears to be a key regulator of this functional switch, playing distinct roles in modulating the entry of different human coronaviruses (Zhao et al., 2018).

Finally, genetic variants in the *IFITM3* gene are associated with severity in various viral infections, including SARS-CoV-2 infection (Čiučiulkaitė et al., 2023; Unali et al., 2023). Research on single nucleotide polymorphisms in IFITM3, such as rs34481144 and rs12252, was conducted to find their effect on the post-vaccine humoral immune response following SARS-CoV-2 infection (Čiučiulkaitė et al., 2023). rs12252 did not show a significant association with the antibody response in a European population, but the GG genotype of rs34481144 was linked with a lower humoral immune response in individuals who received the BNT162b2 vaccine (Čiučiulkaitė et al., 2023). The study shows that *IFITM3* polymorphism may impact vaccine efficacy and contribute to inter-individual differences in SARS-CoV-2 infection susceptibility and severity.

### 3.3 IFITMs beyond viruses: a universal role in blocking EV-mediated communication

**Figure 3:**
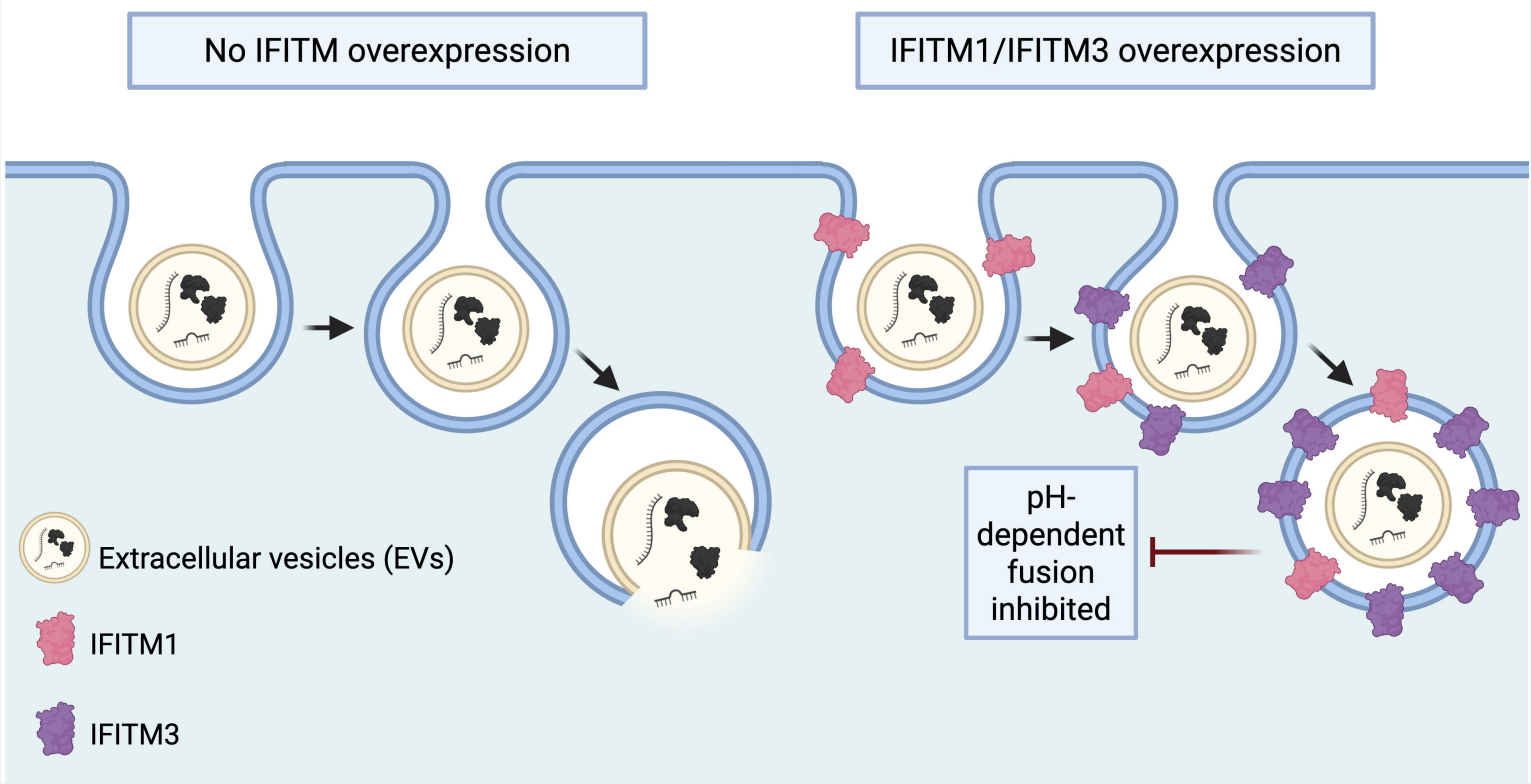
IFITM1 and IFITM3 restrict extracellular vesicle (EV) content delivery. Schematic representation of EV uptake by endocytosis and the subsequent pH-dependent fusion step required for cargo release into the cytoplasm. Left, control condition without IFITM overexpression. Right, IFITM1/IFITM3 overexpression condition. IFITM1 (pink) and IFITM3 (purple) are shown at plasma membrane and internal compartments, with IFITM3 more prominent at endosomal membranes. Although EVs are efficiently taken up by endocytosis, overexpression of IFITM1 and IFITM3 inhibits the pH-dependent fusion step required for cargo release into the cytoplasm, reducing EV-mediated content delivery by over 60% (Created in BioRender; adapted from Bonsergent et al., 2021).

Extracellular vesicles (EVs) are nanoscale lipid-bound vesicles naturally released by living cells, which include exosomes and microvesicles, which mediate intercellular communication by delivering their cargo to recipient cells (Chaudhary et al., 2023). They carry proteins, lipids, and nucleic acids, including microRNAs (miRNAs) that modulate host immune responses. The vesicle structure protects fragile cargo and facilitates its delivery to host cells (Csala et al., 2025; Y. Li et al., 2021). Using this mechanism, parasites broadly exploit EVs to manipulate host defenses and sustain long‐term infection. Beyond viruses, EV–mediated communication is also exploited by helminths (multicellular parasitic worms such as nematodes, trematodes, and cestodes), protozoa (single-celled eukaryotic parasites), and bacteria (prokaryotic pathogens), each of which secrete EVs (Coakley et al., 2016; Mantel and Marti, 2014; Schwechheimer and Kuehn, 2015). Helminths, for example, deliver EVs containing small RNAs, immunoregulatory proteins, and lipids that suppress type-2 immune responses, alter antigen‐presenting cell function, and promote regulatory or anti-inflammatory environments in the host (Drurey and Maizels, 2021; Riera-Ferrer et al., 2024). Interestingly, EV uptake by host cells often mimics viral entry mechanisms, relying on receptor recognition, endocytosis, and subsequent membrane fusion at the plasma or endosomal membrane (Bonsergent et al., 2021; Yu et al., 2023). This similarity raises the possibility that host antiviral mechanisms, especially IFITMs, also restrict EV uptake and fusion. Bonsergent et al. showed that in HEK293T acceptor cells exposed to HeLa cell-derived EVs, IFITM1 and IFITM3 overexpression did not block EV uptake, but instead inhibited EV content delivery to the cytosol by more than 60% (**Figure 3**) (Bonsergent et al., 2021). In cancer models, however, IFITM1 expression has been associated with reduced EV internalization, and gene inactivation restored uptake. Mechanistically, IFITMs alter membrane properties to block fusion, extending their antiviral role to a broader function as “gatekeepers” of intercellular communication (Kelemen et al., 2021).

In contrast to acute infections, chronic parasitic diseases are often associated with the capacity of the pathogen to modulate the host immune system in a systemic and long-term manner to ensure its indefinite survival (Drurey and Maizels, 2021; Gazzinelli-Guimaraes and Nutman, 2018). Because IFITM1 and IFITM3 inhibit EV content delivery to the recipient cell cytosol in mammalian cell systems (Bonsergent et al., 2021), parasite EV signaling provides a useful non-viral framework for considering whether IFITMs might also influence host-pathogen communication beyond virology. However, direct evidence that IFITMs restrict parasite EV uptake or cargo delivery is still lacking. Notably, successful delivery of parasite EV cargo to the host-cell cytosol is likely a prerequisite for effective immunomodulation, as many regulatory RNAs and effector proteins require cytosolic access to alter host signaling pathways. Complex molecular systems for continuous and systemic signaling between parasite and host are likely to have evolved because of this need for stable coexistence. Parasite secretions like excretory/secretory (ES) products and, crucially, EVs, are the main mediators of this communication in host-parasite interactions (Eichenberger et al., 2018; Zhu et al., 2024). This suggests that host factors controlling endosomal escape—such as IFITM3, which localizes to endosomal membranes—may act as critical regulators of whether parasite-derived signals are functionally delivered. In parasitic systems, EVs function as advanced delivery vehicles of effector molecules, such as proteins and regulatory nucleic acids such as miRNAs, between parasites and hosts (Buck et al., 2014; Meningher et al., 2020; Zhong et al., 2024).

In the helminth *Schistosoma mansoni* and protozoan parasites including *Leishmania* spp., EV cargo includes regulatory RNAs and immunomodulatory proteins that alter host-cell signaling and immune differentiation (Meningher et al., 2020; Dong et al., 2021). If chronic infection depends in part on this EV-based inter-kingdom communication, interfering with EV formation, cargo loading, uptake, or delivery represents a plausible therapeutic opportunity (Sánchez-López et al., 2021; Kuipers et al., 2025).

Helminth infections typically induce a Type 2 immune response, characterized by a rise in T helper 2 (Th2) cells, along with a strong regulatory component, allowing chronic infections, rather than sterile immunity. Helminth EVs are central to establishing this balance, for instance delivering regulatory nucleic acids to steer T cell differentiation and inhibit inflammatory pathways (Liu et al., 2019; Zakeri et al., 2018). This process is highly effective; and *Schistosoma* EVs, for example, are efficiently internalized by T helper cells, likely transporting parasitic signals to secondary lymphoid organs and modulating T cell differentiation at its source (Liu et al., 2019; Meningher et al., 2020).

A key effector molecule identified in EV-mediated host modulation is the schistosomal miRNA miR-10, which was shown to post-transcriptionally silence host genes such as MAP3K7 (also known as TAK1), a pivotal signaling molecule required for activating the NF-κB pathway. Upon exposure to schistosomal EVs, silencing of MAP3K7 inhibits NF-κB activity, a pathway essential for robust T cell activation and stable Th2 lineage commitment (Meningher et al., 2020). This mechanism likely reduces the quality and effector function of the anti-parasitic Th2 response. More broadly, schistosome infection and/or schistosome-derived products can drive systemic modulation of T helper cells, including reduced Th2 fidelity, potentially via altered master regulators (e.g., GATA3) and increased immune checkpoint pathways (e.g., PD-L1). Whether parasite EVs alone are sufficient to induce these systemic effects remains to be fully resolved (Yuan et al., 2021).

Protozoan parasites such as *Leishmania* spp. also release EVs containing immunomodulatory cargo, but the specific contribution of EV-delivered factors relative to the broader secretome remains less well resolved than in the schistosome example above (Atayde et al., 2019; Zauli et al., 2023).

Together, these examples indicate that parasite EVs can reshape host immunity through defined cargo molecules (Atayde et al., 2019; Meningher et al., 2020). Although direct evidence linking IFITM family members to parasite EV restriction is currently lacking, several mechanistic parallels suggest potential points of intersection. IFITMs localize to endosomal membranes (Suddala et al., 2019; Zhao et al., 2018) and alter membrane properties (Guo et al., 2021), which could plausibly influence EV uptake or fusion within the endocytic pathway (Desai et al., 2014). Accordingly, the extent to which parasite EV cargo reaches the host cytosol—and thus the magnitude of host immunomodulation and progression toward chronic infection—may depend, at least in part, on host IFITM expression levels, particularly IFITM3 within endosomal compartments. In addition, IFITM3 has been shown to regulate innate immune signaling indirectly through interactions with Nogo-B, modulating Toll-like receptor (TLR) trafficking and downstream NF-κB–dependent cytokine production (Clement et al., 2022). This raises the possibility that IFITMs could influence EV-mediated suppression of MAP3K7 (TAK1)-dependent NF-κB signaling by altering receptor localization or signaling dynamics, although this remains to be experimentally tested (Gomez et al., 2009).

## 4 The host–pathogen arms race – IFITM function in a broader context

### 4.1 Direct pathogen restriction: a first line of defense

IFITM proteins inhibit viral entry by remodeling local membranes, and a common biophysical model proposes increased membrane order and positive spontaneous curvature across the IFITM family, although this mechanism has been most directly supported for IFITM3 (Desai et al., 2014; Li et al., 2013; Suddala et al., 2019).

In reconstituted membrane systems, the IFITM3 amphipathic helix can remodel bilayers and alter curvature in a lipid-dependent manner, with effects that can differ depending on membrane composition (including cholesterol content) (Desai et al., 2014; Guo et al., 2021; Wang et al., 2024). In cells, the dominant antiviral phenotype is consistent with increased lipid order and an inhibition of progression to productive fusion, often described as an energetically unfavorable elasticity/curvature landscape that prevents fusion-pore formation or expansion (Markin and Albanesi, 2002; Li et al., 2013; Suddala et al., 2019; Guo et al., 2021; Klein et al., 2023).

Studies examining the antimicrobial spectrum of IFITM proteins have investigated whether their restriction extends beyond viruses to other intracellular pathogens. Researchers used *Ifitm3*-knockout mice, which are highly susceptible to viral infections. They tested these mice for their vulnerability to the parasite *Plasmodium berghei* ANKA, a model of experimental cerebral malaria. No differences were observed between knockout and wild-type mice in infection rate, disease progression, parasite burden, or cytokine responses, suggesting IFITM3 does not protect against *Plasmodium* (Everitt et al., 2013).

These findings highlight the mechanistic specificity of IFITM proteins: their antiviral activity results from altering host membrane properties to block viral fusion. Although *Plasmodium* interacts with host membranes during invasion, it forms a parasitophorous vacuole through membrane invagination (Garten and Beck, 2021; Geoghegan et al., 2021). This process does not involve direct membrane fusion. Direct membrane fusion is the key step that IFITM restricts (Li et al., 2013; Friedlová et al., 2022). This mechanism is irrelevant to pathogens like *Plasmodium*, which invade by forming a parasitophorous vacuole rather than fusing directly with host membranes (Koch and Baum, 2016). The outcome emphasizes an important principle of innate immunity: IFN responses are wide-ranging, but individual IFN-stimulated genes (ISGs) have specific roles. Therefore, IFITMs do not seem to be general antimicrobial agents; instead, they are specialized antiviral effectors that counter the entry methods of enveloped viruses.

### 4.2 Pathogen evasion and subversion of the IFITM gate

Given their wide antiviral reach, IFITMs probably exert strong selection on pathogens, which respond by neutralizing IFITMs or attenuating the IFN pathways that induce them. Viruses like HIV-1 are examples of the first strategy, while some protozoan parasites use the second (Ray et al., 2000; Diamond and Farzan, 2013; Lüder, 2024; Wang et al., 2024).

Lentiviruses have developed accessory proteins to evade host restriction and innate immunity, often hijacking the host ubiquitin–proteasome pathway to target restriction factors for degradation. For example, HIV-1 Vpu recruits an E3 ligase complex to ubiquitinate tetherin/BST2, directing it toward lysosomal degradation and ensuring efficient virion release (Mitchell et al., 2009; Jia et al., 2014; Seissler et al., 2017). Vif likewise hijacks a host E3 ubiquitin ligase complex to degrade APOBEC3G (Kouno et al., 2023). Nef weakens antiviral defenses by removing IFITM proteins from lipid rafts and EVs, preventing their incorporation into budding virions and thereby limiting antiviral transfer between cells (da Silva-Januário et al., 2023). Env also directly interacts with IFITMs in virus-producing cells, impairing Env processing and incorporation into virions (Yu et al., 2015). Notably, transmitted/founder viruses are more resistant to IFITM restriction, whereas chronic-phase variants, often bearing immune-escape mutations in Env, tend to become more sensitive (Foster et al., 2016). This multifaceted strategy highlights how HIV-1 accessory proteins (Vpu, Nef, Vif, and Env) cooperate to overcome the host’s layered antiviral defenses (Compton et al., 2014; Usami et al., 2015).

Whether pathogen-driven suppression of IFN signaling meaningfully alters IFITM1–3 expression or function during parasitic infection remains unresolved.

The function of IFITM proteins relies on careful regulation of their expression, involving both transcriptional and post-transcriptional mechanisms. Notably, IFITM4P, a long noncoding RNA (ncRNA) transcribed from the IFITM3 pseudogene, acts as a competitive endogenous RNA (ceRNA) by binding miR-24-3p and preserving IFITM1, IFITM2, and IFITM3 mRNAs from degradation (Friedlová et al., 2022; Xiao et al., 2021). These findings suggest that pathogens may evolve miRNAs or other ncRNAs to modulate host immunity broadly, with the potential to directly or indirectly target *IFITM* transcripts and impair innate defenses. Supporting this concept, multiple pathogens encode or induce ncRNAs that reshape host immune pathways. For example, *Herpesvirus saimiri* produces HSUR RNAs that destabilize host miRNAs such as miR-27a (Cazalla and Steitz, 2010), while Epstein–Barr virus encodes miRNAs that dampen type I IFN responses in infected B cells (Bouvet et al., 2021). RNA viruses such as SARS-CoV-2 may also generate small RNAs that act as miRNA sponges and disrupt host regulatory networks (Neeb et al., 2022; Ruivinho and Gama-Carvalho, 2023). Beyond viruses, helminth-derived miRNAs, including miR-71, have been shown to downregulate *Ifitm3* expression in CD4^+^ T cells, albeit indirectly (Soichot et al., 2022). Together, these observations suggest that ncRNA-mediated modulation of host pathways may extend to IFITM regulation and warrants further investigation.

### 4.3 Bacterial infections and context-dependent effects

Studies on bacterial infection have shown that IFITM1, IFITM2 and IFITM3 can restrict *Mycobacterium tuberculosis* in human cells (Ranjbar et al., 2015). Ranjbar et al. demonstrated that infection with *M. tuberculosis* together with stimulation by TLR2 and TLR4 ligands and pro-inflammatory cytokines induces expression of *IFITM1*, -2, and -3. Knockdown of all three genes promotes intracellular bacterial growth, whereas *IFITM3* overexpression drives colocalization with early and especially late *M. tuberculosis* phagosomes, enhancing endosomal and late endosomal acidification and thereby supporting bacterial control (Ranjbar et al., 2015).

In addition, a human genetic study linked a promoter polymorphism in *IFITM3* (rs3888188) to susceptibility to pediatric tuberculosis in Han Chinese children. The G allele, associated with lower promoter activity and reduced expression, correlated with increased disease risk (Shen et al., 2013).

The effects of IFITM proteins are not universally protective and are strongly context-dependent. In macrophages, type I IFN induces IFITM3, which suppresses phagosomal proteolysis of *Listeria monocytogenes* virulence factors ActA and LLO. This reduces bacterial killing and promotes cell-to-cell spread. Mice and cells lacking *Ifitm3* show improved bacterial clearance, increased recruitment of immune cells and reduced dissemination (Tan et al., 2021).

It is also important to note that an earlier mouse study examining infection with the laboratory strain *M. tuberculosis* H37Rv reported no difference in lung bacterial burden between wild type and *Ifitm3* knockout animals during the first thirty days after aerosol infection (Everitt et al., 2013). These findings are consistent with the idea that the outcome may depend on the time point examined, the strain used or redundancy among the murine *Ifitm* genes rather than indicating the absence of a role.

## 5 Broader implications and future directions

### 5.1 Noncanonical functions in cancer and adaptive immune signaling

Recent studies show that IFITM1, IFITM2, and IFITM3 contribute not only to innate immunity but also to cancer biology. They are frequently overexpressed in a wide range of cancers, including colorectal, gastric, breast, prostate, lung, liver, and gliomas, where high expression is typically linked to poor prognosis and aggressive disease (Rajapaksa et al., 2020; Friedlová et al., 2022; Gómez-Herranz et al., 2023).

IFITMs actively contribute to several hallmarks of cancer, including enhanced proliferation, migration, invasion, metastasis, angiogenesis, therapeutic resistance, and the development of stem-like phenotypes. For example, IFITM3 promotes stemness and metastasis in gastric cancer (Chu et al., 2022), and facilitates angiogenesis in glioblastoma via JAK2/STAT3-induced bFGF secretion (Cui et al., 2025). IFITM1 promotes immune evasion by suppressing natural killer cell–mediated killing of gastric cancers (Sakamoto et al., 2020). These activities are frequently the consequence of IFITM overexpression in response to inflammatory stimuli such as IFNs, TGF-β, WNT/β-catenin. This connection links chronic inflammation to tumor progression (Rajapaksa et al., 2020; Friedlová et al., 2022).

Recent work shows that IFITM1 expression enhances the efficacy of programmed cell death protein 1 (PD-1) immune checkpoint blockade and, in combination with an IFITM1-overexpressing oncolytic virus, suppresses brain metastases in mouse models (She et al., 2023). High IFITM3 expression is associated with longer, progression-free survival in patients with small cell lung cancer treated with PD-1 immunotherapy, but not with combined chemo-immunotherapy (Cui et al., 2025). IFITMs act as both prognostic biomarkers and as modulators of therapeutic responses. This dual role shows their complex yet critical significance in clinical oncology.

In adaptive immunity, IFITMs help determine the fate of CD4^+^ T helper cells. Mice lacking the IFITM family (*Ifitm*Del^^-^/^-^^) show a bias toward Th1 differentiation and reduced Th2 responses both *in vitro* and in murine models of allergic airway disease. These mice also exhibit milder Th2-immunopathology, with lower IL-4 and IL-13 expression (Yánez et al., 2019).

Recent studies show that IFITM proteins extend their impact well beyond immune defense. They play pivotal roles in cellular differentiation and embryonic development (Friedlová et al., 2022). During myogenesis, IFITM1, 2, and 3 show significantly higher expression in C2C12 myoblasts, a mouse skeletal muscle cell line commonly used as a model of muscle differentiation. This increase continues as differentiation progresses and is associated with elevated levels of myogenic markers, including MyoD, myogenin, Myf5, and desmin. Knocking down each Ifitm individually disrupts myotube formation and reduces expression of these differentiation factors. Moreover, IFITM1 and 3 interact with key cytoskeletal and sarcomeric proteins (such as actin, myosin, vimentin, and desmin), suggesting that they help scaffold the molecular machinery required for myogenesis (Zhang et al., 2023).

The specialized IFITM5 (BRIL) protein plays a role in skeletal biology. Mice lacking IFITM5 show only minor skeletal changes under normal conditions. In contrast, the c.-14C>T mutation in the *Ifitm5* 5′ UTR causes osteogenesis imperfecta type V via a mechanism that alters osteoblast differentiation and bone formation (Cho et al., 2012; Lietman et al., 2015).

Overall, these functions point to a common theme: IFITM family members help interpret cellular signals that shape cell fate. Beyond their antiviral activity, they participate in processes such as myoblast fusion, T cell immunity, and osteogenic differentiation, highlighting broader developmental and regulatory roles.

### 5.2 A unified view and unanswered questions

The multifunctional nature of IFITM proteins shows how they evolved under selective pressure from viruses. The IR-*IFITM* genes (*IFITM1–3*) show evidence of lineage-specific gene duplications and positive selection in primates and rodents which fits with the idea of an evolutionary arms race with viruses (Zhang et al., 2012; Friedlová et al., 2022). Although experimental evidence is limited, the strong induction of IFITMs by interferons during infections suggests a likely but as yet unexplored role in antiparasitic defense (Zhao et al., 2018). From an evolutionary perspective, the same molecular features that confer strong antiviral activity to IFITMs, such as membrane remodeling and participation in key cellular pathways, can also lead to pathological outcomes. For instance, in cancer metastasis when their regulation is disrupted (Zhao et al., 2018; Friedlová et al., 2022), IFITMs exemplify an evolutionary trade-off: they provide robust defense but can become liabilities when co-opted or dysregulated.

Although significant progress has been made in understanding IFITM proteins, some fundamental questions remain unanswered. The exact mechanisms by which IFITMs inhibit viral infection are still incompletely understood: one model proposes that they rigidify membranes and block hemifusion, the lipid-mixing step preceding fusion (Li et al., 2013). Another suggests that IFITM3 disrupts the VAPA–OSBP interaction, leading to cholesterol accumulation in endosomal compartments and thereby inhibiting viral fusion (Amini-Bavil-Olyaee et al., 2013; Liao et al., 2019).

### 5.3 Future research directions

The membrane topology of IFITMs remains debated. While some studies support a dual transmembrane or mixed-topology “L-shape” model, with a cytosolic N-terminus and an extracellular or luminal C-terminus, other evidence favors a hairpin-like structure inserted into the inner leaflet, with both termini remaining in the cytosol (Weston et al., 2014; Friedlová et al., 2022). The specificity of IFITM-mediated restriction depends on both the virus and the subcellular localization of the proteins. For instance, IFITM1 is relatively more potent against certain enveloped viruses such as filoviruses, whereas IFITM3 is particularly effective against IAV, likely reflecting differences in localization and viral entry routes (Huang et al., 2011). In cancer, IFITM1 shows context-dependent effects: in some settings, it enhances IFN-γ–mediated growth suppression through p53 stabilization and activation (Yang et al., 2007), while in others, high expression correlates with tumor aggressiveness, promoting proliferation and metastasis (Ogony et al., 2016; Zheng et al., 2017).

Taken together, unresolved questions about IFITM mechanisms, structure, specificity, and physiological functions highlight key gaps in our understanding. These controversies define the current frontier and point to priorities for future research.

Besides their traditional roles in antiviral immunity, the function of IFITM proteins in parasitic infections remains a major gap in the field (Friedlová et al., 2022). Protozoan pathogens such as *Plasmodium*, *Toxoplasma*, *Leishmania*, and *Trypanosoma* spp. elicit strong Type I and/or Type II IFN responses in host cells (Beiting, 2014; Deng et al., 2023). Transcriptomic analyses of *Plasmodium*-infected livers show strong IFN-inducible gene expression. This includes upregulation of ISGs such as *Ifitm1*, supporting the idea that IFITMs are likely induced during parasitic infections (Hildebrandt et al., 2024). However, the functional roles of IFITM1–3 in this process are still largely unexplored. Although IFITM activity has been examined in limited parasitic models, existing evidence and the availability of knockout models make this an important avenue for future research. It will be important to investigate whether IFITMs influence parasite infection in organism-specific contexts, for example by modulating host immune signaling or altering EV-mediated communication, rather than assuming a common mechanism across diverse parasites (MacMicking, 2012; Friedlová et al., 2022).

IFITM proteins are considered attractive therapeutic targets in both infectious diseases and cancer, owing to their central role in viral restriction and tumor progression, and their potential therapeutic relevance. Enhancing IFITM activity represents a promising antiviral strategy, offering broad-spectrum protection with reduced risk of viral resistance. Although specific activators for IFITMs have not yet been developed, the concept appears promising. For example, amphotericin B has been shown to counteract IFITM3-mediated restriction of IAV and increase viral replication *in vitro* and in animal models (Lin et al., 2013). Conversely, in several cancers, inhibiting IFITM function holds therapeutic promise, as IFITM overexpression can drive metastasis and invasion. The consequences of IFITM antagonism, however, are likely to be virus-dependent. For viruses that are restricted by IFITMs, chemical antagonism could reverse antiviral activity and restore entry. In contrast, for viruses such as hCoV-OC43 that exploit IFITMs for infection, disrupting IFITM localization would be expected to reduce, rather than enhance, viral entry (Zhao et al., 2018; Xie et al., 2023). Cyclosporine A, for example, rapidly relocates IFITM1 and IFITM3 from their functional membrane sites to the Golgi apparatus, illustrating that chemical antagonism of IFITMs is feasible (Prikryl et al., 2023). Translating these strategies into safe clinical treatments remains a major challenge. IFITMs are also critical for T cell differentiation, development, and tissue homeostasis, meaning systemic manipulation could cause significant side effects. Future therapeutic progress will therefore likely depend on highly targeted approaches, such as tissue-specific delivery systems or modulators that can distinguish between physiological and pathological IFITM functions.
